# Altered Adipose-Derived Stem Cell Characteristics in Macrodactyly

**DOI:** 10.1038/s41598-017-11666-3

**Published:** 2017-09-11

**Authors:** Xi Yang, Yongkang Jiang, Gang Han, Yuan Shi, Shengbo Zhou, Feng Ni, Bin Wang

**Affiliations:** Department of Plastic and Reconstructive Surgery, Shanghai 9th People’s Hospital, Shanghai Jiaotong University School of Medicine, Shanghai, 200011 P.R. China

## Abstract

Macrodactyly is a congenital disease characterized by aggressive overgrowth of adipose tissue in digits or limbs frequently accompanied with hyperostosis and nerve enlargement; its pathological mechanism is poorly understood. Adipose-derived stem cells (ASCs) have been extensively studied in tissue engineering and regenerative medicine as an ideal alternative substitute for bone marrow-derived mesenchymal stem cells (BM-MSCs), but their pathological role is largely unknown. In this study, ASCs from macrodactyly adipose tissues (Mac-ASCs) were isolated and compared to ASCs derived from the normal abdominal subcutaneous adipose tissue (Sat-ASCs) for cell morphology, surface marker expression, proliferation rate, and tri-lineage differentiation potential. Despite similar cell morphology and cell surface marker expression, Mac-ASCs showed higher cell proportion in the S phase and increased proliferation compared with Sat-ASCs. Moreover, osteogenic and chondrogenic differentiation capacities were enhanced in Mac-ASCs, with reduced adipogenic potential. In addition, the expression levels of adipogenic genes were lower in undifferentiated Mac-ASCs than in Sat-ASCs. These findings unraveled enhanced proliferation activity, a regression in the differentiation stage, and greater potentiality of ASCs in macrodactyly, which could contribute to hyperostosis and nerve enlargement in addition to adipose tissue overgrowth in patients.

## Introduction

**Figure 1:**
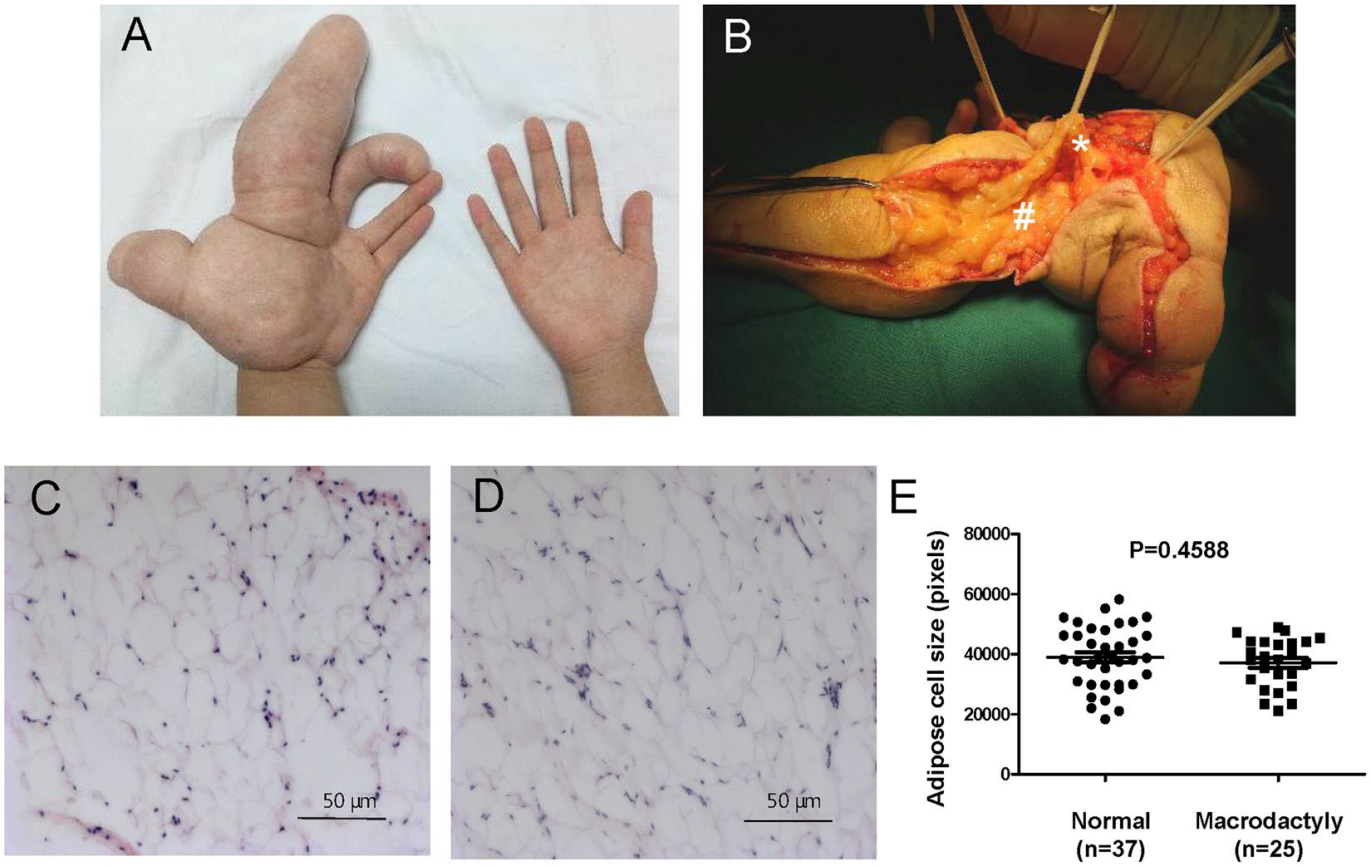
Overgrowth but normal histology of the adipose tissue in macrodactyly. (**A**) Extremely overgrown left hand compared to the unaffected right hand in a patient with macrodactyly. (**B**) Excessive adipose tissue (#) and enlarged digital nerve (*) are shown in defatting surgery of macrodactyly. (**C** and **D**) H&E staining of abdominal subcutaneous adipose tissue **(C)** and adipose tissue in macrodactyly **(D)**. Scale bars, 50 μm. (**E**) The adipocytes size in six random fields, each from three macrodactyly samples and three controls, were measured and quantified by using the ImagePro software (Version 4.0 Analytik, Germany). Unpaired t-test was applied to assess the significance of difference between the two groups.

Macrodactyly is a rare extremity congenital anomaly with the clinical manifestation of aggressive overgrowth of adipose tissue in digits or limbs, with or without hyperostosis and nerve enlargement (Fig. 1A and B). This disorder seriously influences limb function and appearance, especially when the whole limb or even half of the body is affected. Amputation is considered the primary choice for treatment of macrodactyly, when overgrowth grossly interferes with function^1^. Barsky^2^ and Tsuge^3^ refined treatment options to maintain aesthetics and function, but recurrence frequently occurs. Despite abundant phenotypical descriptions^4^, the mechanism of macrodactyly remains poorly understood.

The normal adipose tissue consists of more than 90% of mature adipocytes and a stromal vascular fraction (SVF), which is composed of preadipocytes, fibroblasts, endothelial cells, resident immune cells, and a population of multipotent stem cells, namely adipose-derived stem cells (ASCs)^5^. Isolated ASCs have considerable proliferation capacity and can differentiate along adipogenic, osteogenic, chondrogenic, and other pathways *in vitro* ^6^. ASCs derived from subcutaneous adipose tissues of different anatomic regions display comparable proliferation, adipogenic, osteogenic, and chondrogenic differentiation potentials^7^. They have been extensively studied in tissue engineering and regenerative medicine as an ideal substitute for bone marrow-derived mesenchymal stem cells (BM-MSCs)^5, 8^. However, the role of ASCs in pathological conditions remains largely unknown.

To explore the pathogenesis of macrodactyly, ASCs from macrodactyly adipose tissue (Mac-ASCs) were isolated and compared to those isolated from normal subcutaneous adipose tissue (Sat-ASCs). Despite similar cell morphology and surface marker expression, Mac-ASCs had higher cell proportion in the S phase and increased proliferative activity compared with Sat-ASCs. Moreover, osteogenic and chondrogenic differentiation capacities were enhanced in Mac-ASCs, while their adipogenic potential was actually reduced. These altered characteristics of Mac-ASCs could contribute to the frequently observed hyperostosis and nerve enlargement accompanying adipose tissue overgrowth in macrodactyly. These findings unraveled a potential pathological relevance between altered ASC characteristics and macrodactyly.

## Results

### The adipose tissue from macrodactyly has normal histology

To assess whether there was histological abnormalities in the adipose tissue from macrodactyly, we compared H&E stained adipose tissues from macrodactyly and normal abdominal subcutaneous tissues. The size of the adipocytes showed no significant differences between the two groups (Fig. 1C–E), indicating a normal histology for the adipose tissue in macrodactyly.

### Mac-ASCs express common ASC cell surface markers

**Figure 2:**
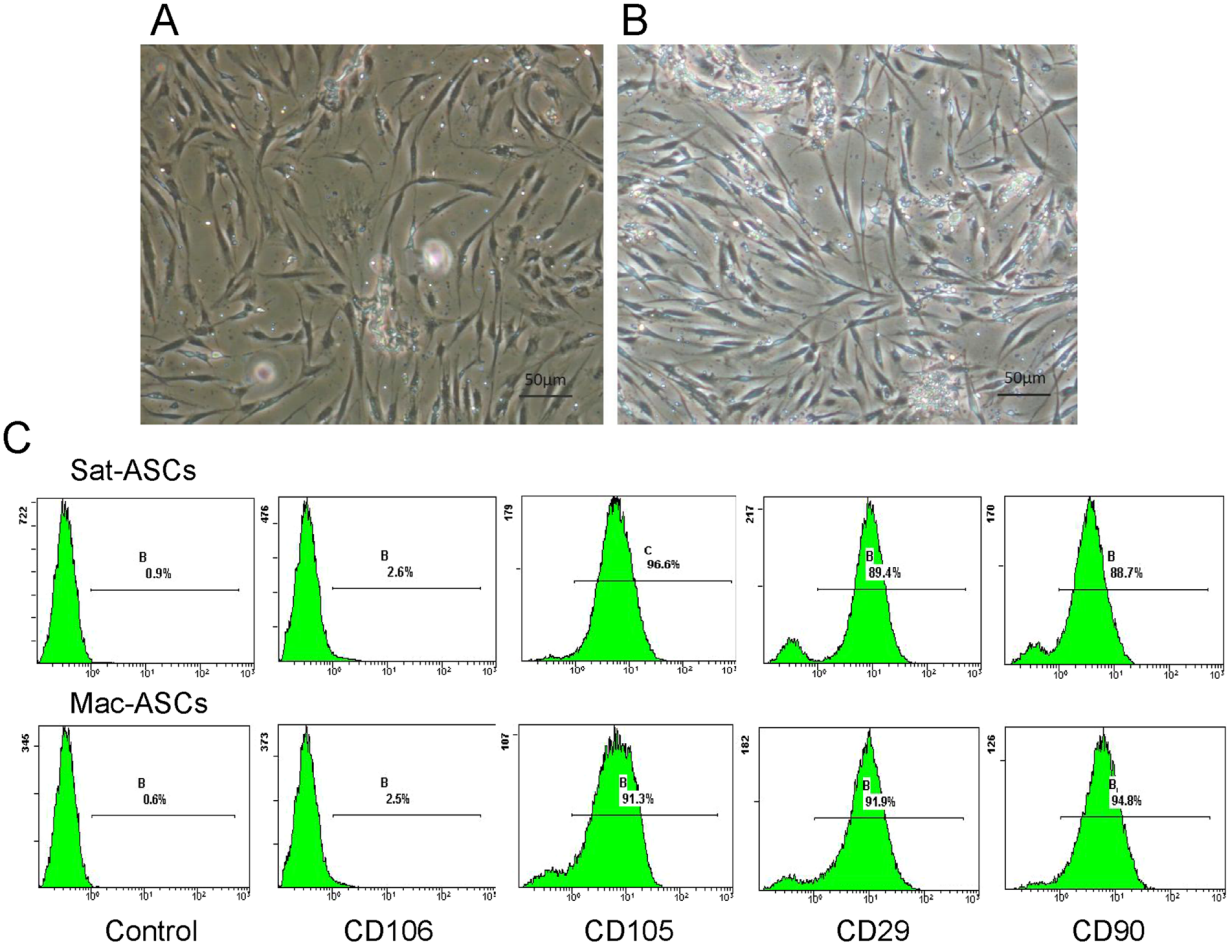
Similar cell morphology and surface marker expression in Sat-ASCs and Mac-ASCs. (**A** and **B**) Cell morphology of Sat-ASCs **(A)** and Mac-ASCs **(B)** at culture day 5. Scale bars, 100 μm. (**C**) Sat-ASCs (upper panel) and Mac-ASCs (lower panel) were stained with antibodies against the ASC- specific markers CD105, CD29, and CD90, and the BM-MSC-specific marker CD106, and analysed by flow cytometry.

To evaluate whether there was any anomaly of ASCs in macrodactyly, we isolated ASCs from adipose tissues of macrodactyly. ASCs from normal abdominal subcutaneous adipose tissues were also obtained for comparison. Interestingly, Mac-ASCs and Sat-ASCs both displayed a spindle shape in the preliminary culture and subcultures, and cell morphology showed no significant change at up to five passages (Fig. 2A and B). Next, the expression of cell surface markers was assessed in Mac-ASCs and Sat-ASCs. While mock staining showed no signal, up to 90% cells in both Mac-ASC and Sat-ASC groups showed positive staining for the ASC-specific cell surface markers CD105, CD29, and CD90, but the cells were negative for the BM-MSC-specific cell surface marker CD106 (Fig. 2C), consistent with previous findings^9^. These results suggested that there is no differences between the two groups concerning these markers.

### Mac-ASCs show enhanced cell proliferation

**Figure 3:**
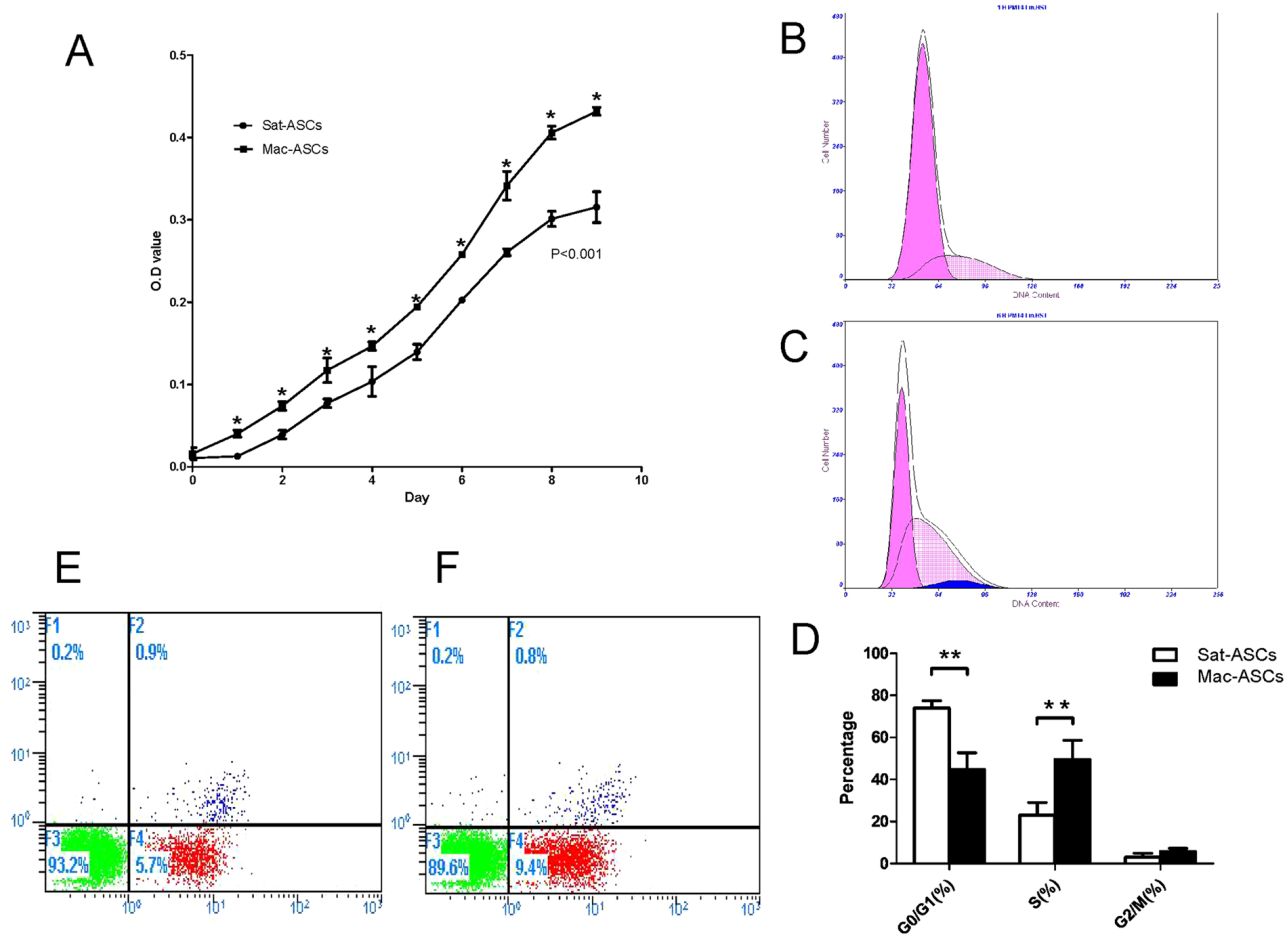
Enhanced proliferative activity of Mac-ASCs. (**A**) A total of 1000 cells (Sat- or Mac-ASCs) were seeded in a 96-well plate, and the MTT assay was performed at different time points. The growth curves of the two cell lines showed significant differences (P < 0.001). (**B** and **C**) Sat- **(B)** and Mac- **(C)** ASCs were stained with PI and analyzed by flow cytometry for cell cycle. Pink, gridded, and blue shaded peaks represent the G0/G1, S, and G2/M phases, respectively. (**D**) Quantification of cell proportions in the G0/G1, S, and G2/M phases in Sat- and Mac-ASCs. (**E** and **F**) Sat- **(E)** and Mac- **(F)** ASCs were co-stained with Annexin V and PI, and analyzed by flow cytometry for cell apoptosis. No difference was observed between Mac-ASCs and Sat-ASCs.

To assess the proliferative activity of Mac-ASCs, cells were seeded in 96-well plates and evaluated by the MTT assay at different time points. The growth curves showed that Mac-ASCs had a significantly higher proliferation rate compared with Sat-ASCs (Fig. 3A). To explore the mechanism underlying the differential proliferation rates, cell cycle distribution and apoptosis were assessed by flow cytometry. Mac-ASCs showed increased cell proportion in the S phase and decreased G0/G1 population compared with Sat-ASCs. Cell proportion in the G2/M phase were similar between Mac-ASCs and Sat-ASCs (Fig. 3B–D). Moreover, the cell proportions in both early and late apoptosis were similar in Mac-ASCs and Sat-ASCs (Fig. 3E and F). Thus, Mac-ASCs had increased cell proportion in the S phase and enhanced proliferation.

### Altered differentiation potential of Mac-ASCs

**Figure 4:**
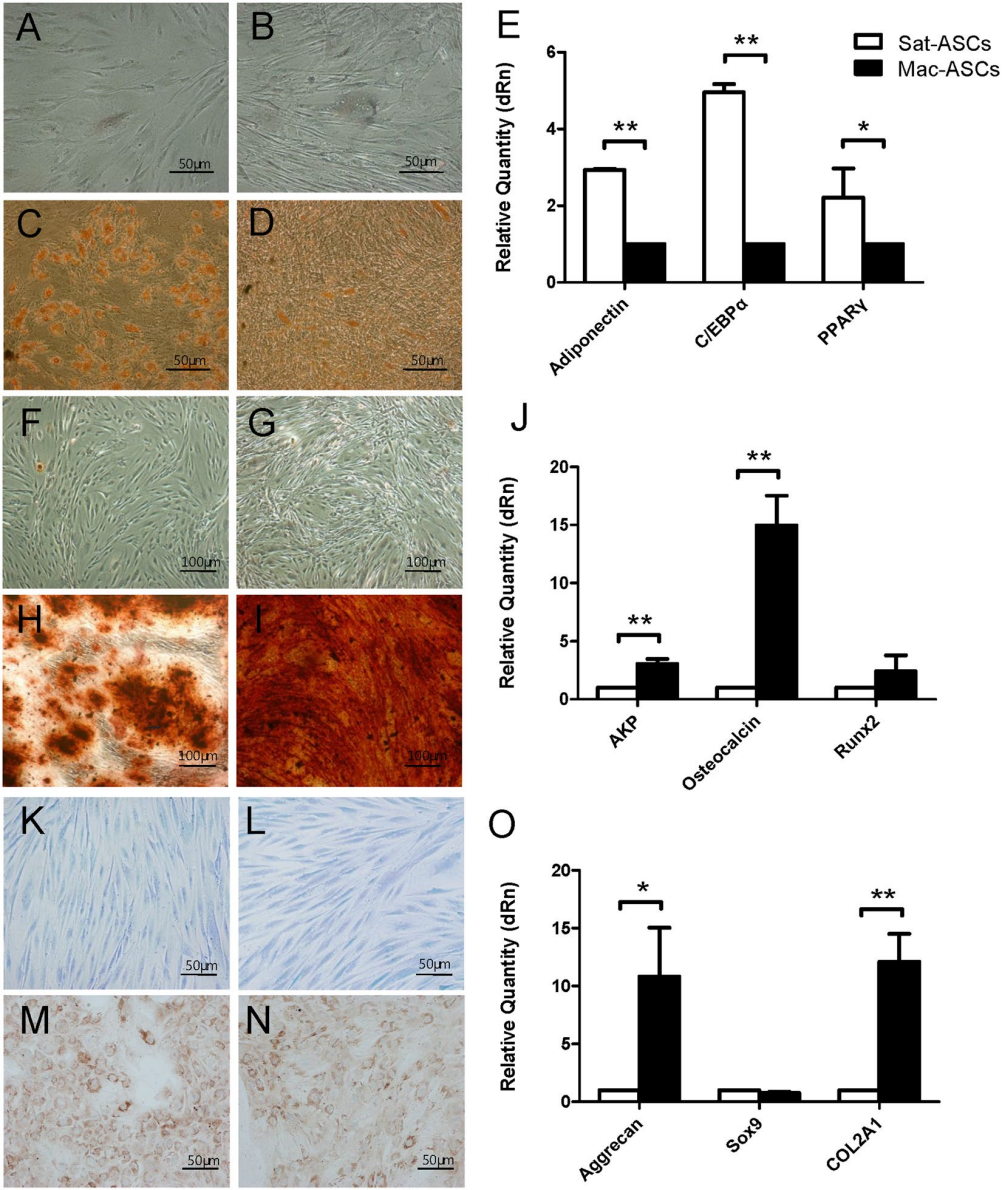
Altered multi-lineage differentiation potential of Mac-ASCs. (**A**–**D**) Uninduced Sat- **(A)** and Mac- **(B)** ASCs, or adipogenic-induced Sat- **(C)** and Mac- **(D)** ASCs were stained with Oil Red. Lipid droplets were stained by Oil Red O, and appeared orange in the cytosol. Scale bars, 50 μm. (**E**) qRT-PCR analysis of the adipogenic genes Adiponectin, C/EBPα and PPARγ in adipogenic-induced Sat- and Mac-ASCs. (**F**–**I**) Uninduced Sat- **(F)** and Mac- **(G)** ASCs, or osteogenic-induced Sat- **(H)** and Mac- **(I)** ASCs were stained with Alizarin Red. Bone nodules were stained by Alizarin Red and appeared red in culture. Scale bars, 100 μm. (**J**) qRT-PCR analysis of the osteogenic genes AKP, Osteocalcin and RUNX2 in osteogenic-induced Sat- and Mac-ASCs. (**K**–**N**) Uninduced Sat- **(K)** and Mac- **(L)** ASCs, or chondrogenic-induced Sat- **(M)** and Mac- **(N)** ASCs were stained with antibody against Collagen II and counterstained with hematoxylin. Collagen II signals appeared brown in the cytosol. Scale bars, 50 μm. (**O**) qRT-PCR analysis of the chondrogenic genes Aggrecan, Sox9 and Col2A1 in chondrogenic-induced Sat- and Mac-ASCs.

We then assessed the differences in multi-linage differentiation potential between Mac- and Sat-ASCs. Adipogenic induction was successful because a significant accumulation of lipid droplets stained by Oil Red O was observed in induced Sat- (Fig. 4C) and Mac-ASCs (Fig. 4D), but not in non-induced counterparts (Fig. 4A and B, respectively). However, the lipid droplets were much less intense and smaller in Mac-ASCs (Fig. 4D) than in Sat-ASCs (Fig. 4B) after induction. To confirm the reduced adipogenic differentiation of Mac-ASCs, the mRNA expression levels of Adiponectin, C/EBPα, and PPARγ were assessed by qRT-PCR; these genes are late, medium, and early white adipose tissue markers, respectively^10–12^. Consistent with the phenotypic observation, the mRNA levels of all three genes were significantly lower in Mac-ASCs than in Sat-ASCs after adipogenic induction (Fig. 4E).

Next, we performed osteogenic differentiation experiments. After 14 days of osteogenic differentiation, the cells were stained with Alizarin Red, a specific marker of bone nodules. Intense Alizarin Red staining was observed in induced Sat-ASCs (Fig. 4H) but not in non-induced counterparts (Fig. 4F), indicating an efficient osteogenic differentiation. Unexpectedly, induced Mac-ASCs exhibited even much stronger Alizarin Red staining (Fig. 4I), suggesting enhanced osteogenic differentiation compared with Sat-ASCs. Consistently, qRT-PCR analysis showed a dramatically increased mRNA expression of the osteogenic marker osteocalcin^13^ and moderately increased AKP and RUNX2 mRNA levels^13^, in differentiated Mac-ASCs compared with the levels obtained for Sat-ASCs (Fig. 4J).

Then, we assessed whether chondrogenic differentiation potential was also affected in Mac-ASCs. The protein levels of Collagen II, a chondrogenic differentiation marker, were comparable between induced Mac-ASCs and Sat-ASCs as indicated by IHC (Fig. 4K–N). However, qRT-PCR analysis revealed higher mRNA expression levels of Col2A1 and Aggrecan, another chondrogenic marker^13^ in induced Mac-ASCs than in the Sat-ASC group (Fig. 4O). The expression of another chondrogenic marker, Sox9^13^ was unchanged (Fig. 4O).

### Undifferentiated Mac-ASCs show reduced adipogenic gene expression

**Figure 5:**
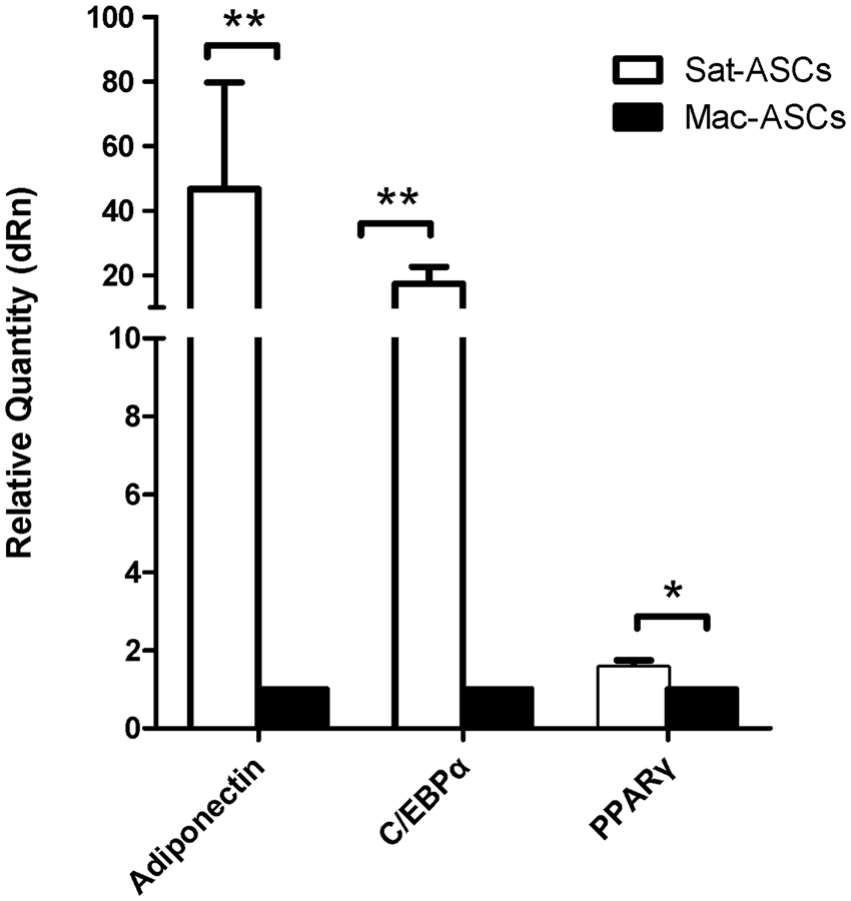
Reduced adipogenic gene expression in undifferentiated Mac-ASCs. qRT-PCR analysis of the adipogenic genes Adiponectin, C/EBPα and PPARγ in undifferentiated Sat- and Mac-ASCs was performed.

The reduced adipogenic, and elevated osteogenic and chondrogenic differentiation potentials of Mac-ASCs may reflect a regression in the adipogenic differentiation commitment. To test this hypothesis, the expression levels of adipogenic genes in undifferentiated Mac-ASCs were evaluated. qRT-PCR analysis revealed that the mRNA expression levels of Adiponectin, C/EBPα, and PPARγ were indeed significantly lower in undifferentiated Mac-ASCs than in Sat-ASCs (Fig. 5), suggesting that Mac-ASCs were less adipogenic differentiation-committed. These findings indicated a regression in the differentiation stage where the cells came to have greater potentiality in Mac-ASCs.

## Discussion

Since their first identification in 2001, ASCs have been extensively studied as an ideal substitute for BM-MSCs in tissue engineering and regenerative medicine. However, their pathological role remains elusive. Macrodactyly is a progressive congenital anomaly characterized by aggressive overgrowth of the adipose tissue, frequently accompanied with hyperostosis and nerve enlargement in digits or limbs^1–3^. Its pathological mechanism is poorly understood. In this study, we compared ASCs derived from adipose tissues of macrodactyly and normal abdominal subcutaneous adipose tissues for their properties. Despite similar cellular morphology and cell surface marker expression, Mac-ASCs displayed increased cell proportion in the S phase and enhanced proliferation compared with Sat-ASCs. Meanwhile, elevated osteogenic and chondrogenic, but attenuated adipogenic differentiation potential were found for Mac-ASCs.

Because it is impossible to obtain adipose tissues from normal hands, abdominal subcutaneous adipose tissues obtained in necessary defatting of abdominal skin for grafting operation were selected as normal controls. Human ASCs derived from different anatomic regions have been compared for their proliferative activity and differentiation potential. Schipper *et al*.^14^ reported that ASCs derived from various subcutaneous regions, including the arm, thigh, inguinal region, and abdomen, display comparable proliferation rates and adipogenic differentiation potential. Jurgens *et al*.^15^ demonstrated that ASCs derived from the subcutaneous regions of the abdomen and hip/thigh show comparable proliferation rates, with no significant differences in osteogenic or chondrogenic differentiation potential. Therefore, the altered proliferation activity and differentiation potential of Mac-ASCs are likely not attributable to anatomical differences but reflect the pathological condition of macrodactyly.

The altered differentiation potential of Mac-ASCs was intriguing. Preadipocytes isolated from subcutaneous depots have a higher adipogenic potential than those obtained from omental or mesenteric depots, suggesting that the subcutaneous tissue produces progenitor cells more committed to mature adipocytes^14^. Mac-ASCs displayed enhanced osteogenic and chondrogenic, but reduced adipogenic differentiation potential, suggesting a regression in the adipogenic differentiation commitment of Mac-ASCs. This notion was supported by the decreased expression of adipocyte-specific genes in undifferentiated Mac-ASCs. While tissue overgrowth in macrodactyly is probably a result of elevated ASC proliferation activity, bone hyperostosis and nerve enlargement could be a consequence of the elevated potential of Mac-ASCs. If this is the case, removing ASCs by surgical intervention, such as defatting, could be beneficial in controlling not only the overgrowth of adipose tissue but also the bone hypertrophy and nerve enlargement in the affected digits.

Recent studies have associated macrodactyly with somatic activating mutations of PIK3CA^16, 17^. The latter gene encodes the p110a catalytic subunit of PI3K, an essential component of the RTK-PI3K-AKT signaling pathway that is critical for cellular growth and metabolism^18^. PIK3CA mutations frequently occur in many human cancer types, and have been established as causative and key driver of cancer^19^. Interestingly, the expression of H1047R (one of the most frequent mutations of PIK3CA) in lineage-committed basal or luminal cells of the adult mouse mammary gland evokes cell dedifferentiation into a multipotent state that contributes to tumor heterogeneity^20^. The potential functional impact of PIK3CA mutations on the alteration of Mac-ASC characteristics is of particular interest, and should be addressed in future studies.

## Methods

### Samples

This study was approved by the ethics committee of Shanghai 9th People’s Hospital. Adipose tissue samples were obtained from defatting surgery of macrodactyly. Normal controls were abdominal subcutaneous adipose tissues from necessary defatting of abdominal skin for grafting operation. Informed consent was obtained from all patients. Eight samples from macrodactyly patients (six males and two females, each from the hand or foot) and six samples from normal controls (two males and four females, all from the abdomen) were collected; the donors ranged from 2 to 12 years old.

### H&E staining

The surgically removed adipose tissue samples were fixed in 4% paraformaldehyde, followed by H&E staining, as previously described^21, 22^.

### ASC isolation and culture

ASCs were isolated from adipose tissues of macrodactyly and normal abdominal subcutaneous adipose tissues as described previously^23^. Briefly, the adipose tissue was minced under sterile conditions, and digested with 0.05% Collagenase II (Sigma-Aldrich, St. Louis, USA) in serum-free DMEM (Hyclone, Logan, USA) at 37 °C for 2–3 h. After digestion, the fat tissue was filtered and centrifuged (1500 rpm at 37 °C for 5 min). The cells were re-suspended in low glucose DMEM (Hyclone, Logan, USA) containing 10% FBS (Gibco, Grand Island, USA) and 100 μg/mL penicillin-streptomycin (Gibco, Grand Island, USA), and cultured at 37 °C in a 5% CO_2_ incubator (HEPA class 100, Thermo, Waltham, USA). When the cells became confluent, they were detached with 0.25% trypsin-EDTA (Gibco, Grand Island, USA) and subcultured at the same density for passage.

### Immunostaining of cell surface markers and flow cytometry

Immunostaining was performed as previously described^9, 24^. Briefly, Mac-ASCs and Sat-ASCs (P1) were harvested by centrifugation, washed once with PBS, and re-suspended in PBS. Approximately 10^5^ cells in 1 mL of PBS were incubated with 5 μL of anti-CD106, anti-CD105, anti-CD29, or anti-CD90 antibodies (eBioscience, CA, USA) for 20 min. Then, the cells were washed and re-suspended in 1 mL PBS, and the expression of cell surface markers was analysed by flow cytometry.

### MTT assay

Passage 2 (P2) cells were seeded in triplicate into 96-well plates (1 × 10^3^ cells/well) and incubated for 24 h. Blank controls were set, the medium was changed every 2 to 3 days, and 10 μL of MTT solution (5 mg/mL) (Biotechwell, Shanghai, China) in low glucose DMEM (100 μL) was added every 24 hours. After 4 hours of incubation, the medium was removed, and the purple formazan crystals formed were dissolved in 100 μL DMSO for 10 min. Absorbance was obtained on a microplate reader (5250030 Varioskan Flash, Thermo Scientific Inc.) at 490 nm. The assay was repeated three times.

### Cell cycle analysis

Cell cycle analysis was performed by flow cytometry^25^. Mac-ASCs and Sat-ASCs in logarithmic phase (on the second day according to the MTT assay) were harvested. Next, 10^5^ cells were re-suspended in 0.5 mL of PBS and fixed with 70% ethanol at 4 °C overnight. After washing, cells were incubated with RNase A (20 μg/mL) at 37 °C for 30 min, stained with PI (100 μg/mL, BD Biosciences, USA) for 10 min, and analysed by flow cytometry.

### Apoptosis analysis

Cell apoptosis was assessed using Annexin V-FITC and PI fluorescent staining (Alexa®Fluor 488 Annexin V and PI Kit, no.V13241, Invitrogen, USA). Briefly, Mac-ASCs and Sat-ASCs were collected by centrifugation and washed once with PBS. Approximately 10^5^ cells in 1 mL of PBS were subsequently stained with 5 μL Annexin V-FITC and 1 μL PI for 20 min at room temperature. Blank control, and Annexin V and PI single staining controls were assessed as well. Flow cytometry data were analysed with the ModFit software (version 4.0, Verity Software House Inc., Topsham, ME, USA).

### Adipogenic, osteogenic and chondrogenic differentiation of ASCs

Adipogenic, osteogenic and chondrogenic induction was performed for Mac-ASCs and Sat-ASCs (P1). Cells were harvested and re-suspended in low glucose DMEM, carefully added to each well of a 6-well plate (3000 cells/cm_2_), and allowed to adhere and grow to 60% confluence at 37 °C in presence of 5% CO_2_. The medium was then changed to adipogenic differentiation medium (human no. HUXMD-90031 Cyagen Bioinformatics, Suzhou, China. Medium A: 175 mL basal medium, 20 mL FBS, 2 mL penicillin-streptomycin, 2 mL glutamine, 400 μL insulin, 200 μL IBMX, 200 μL rosiglitazone and 200 μL dexamethasone. Medium B: 175 mL basal medium, 20 mL FBS, 2 mL penicillin-streptomycin, 2 mL glutamine, and 400 μL insulin), osteogenic differentiation medium (DMEM-high glucose [Hyclone, Logan, USA], 10% FBS, 100–100 μg/mL penicillin-streptomycin, 50 μg/mL L-ascorbic acid-2-phosphate, L-glutamine, 10^−7^ M dexamethasone, and 10 mM β-glycerophosphate [Sigma-Aldrich, St. Louis, USA]), or chondrogenic differentiation medium (human no. HUXMD-90041 Cyagen Bioinformatics, Suzhou, China: 194 mL basal medium, 20 μL dexamethasone, 600 μL ascorbate, 2 mL ITS supplement, 200 μL sodium pyruvate, 200 μL proline, and 2 ml transforming growth factor-β3). Fresh medium was replaced every 2 days. The adipogenic and osteogenic differentiation groups were induced for 14 days while the chondrogenic differentiation group was induced for 21 days; non-induction groups were run in parallel in low glucose DMEM containing 10% FBS and 100 μg/mL of each penicillin and streptomycin).

### Histochemical and immunohistochemical staining

Cells in 6-well plates were fixed with 4% paraformaldehyde for 30 min and washed in PBS 3 times. The adipogenic differentiation group was stained with Oil Red O to evaluate lipid droplets. The osteogenic differentiation group was stained with Alizarin Red for calcium deposit assessment. Immunohistochemical (IHC) staining of Collagen II was performed for the chondrogenic differentiation group. For IHC analysis, the samples were blocked with 3% BSA, and anti-Collagen II primary antibody (rabbit Collagen II, ab34712, 1:200, Abcam, Cambridge, UK) was applied and incubated at 4 °C overnight. After three washes in PBS, horseradish peroxidase (HRP) conjugated detection antibody (GAR, K55007, 1:1, Dako, Denmark) was added with diaminobenzidine tetra-hydrochloride (DAB) as the chromogen. Sections were counterstained with hematoxylin. The slides were analyzed under a light microscope (Eclipse 90i, Nikon, Tokyo, Japan).

### RNA extraction and quantitative real-time polymerase chain reaction (qRT-PCR)

As previously described^10^, total RNA was extracted using TRIzol Reagent (Life Technologies, CA, USA) without detaching the cells. Reverse transcription was performed from 2 μg of total RNA, with oligo (dT) and Revert Aid Reverse Transcriptase (Thermo Scientific Inc.) following the manufacturer’s instructions. The mixture was then incubated at 30 °C for 10 min, 42 °C for 60 min, 95 °C for 5 min, and 5 °C for 5 min. cDNA was amplified using Power SYBR Green PCR (TIANGEN) on a Real-time thermal cycler (Biosystems 7500 Fast Real-time PCR System, Life Technologies, CA, USA); each measurement was performed in triplicate. Relative gene expression levels were obtained by the comparative Ct (cycle threshold) method. Adiponectin, C/EBPα, and PPARγ were used to evaluate the adipogenic potential of cells; AKP, Osteocalcin and Runx2 were employed to assess the osteogenic potential; Aggrecan, SOX9 and Col2A1 were used to determine the chondrogenic potential. GAPDH was used as an internal control. The primers used for qPCR are listed in Table 1. The experiments were performed at least in triplicate.

**Table 1:** Primers for qRT-PCR.

Gene	Primer sequence (5′–3′)	Annealing temperature (°C)	Product size (bp)
Adiponectin	Sense: GTGAGAAAGGAGATCCAGGTCTT	60	128
	Antisense: GGCACCTTCTCCAGGTTCTC		
C/EBPα	Sense: CCCTCAGCCTTGTTTGTACT	60	204
	Antisense: AAAATGGTGGTTTAGCAGAGA		
PPARγ	Sense: GCAGTGGGGATGTCTCATAAT	60	115
	Antisense: CAGCGGACTCTGGATTCAG		
Klf4	Sense: CTCGCCTTGCTGATTGTCTATT	58	102
	Antisense: CACCTGAACCCCAAAGTCAAC		
AKP	Sense: TACAAGCACTCCCACTTCATC	58	80
	Antisense: AGACCCAATAGGTAGTCCACAT		
Osteocalcin	Sense: CACCGAGACACCATGAGAGC	58	132
	Antisense: CTGCTTGGACACAAAGGCTGC		
Runx2	Sense: ACAGTAGATGGACCTCGGGA	58	113
	Antisense: ATACTGGGATGAGGAATGCG		
Aggrecan	Sense: AGTATCATCAGTCCCAGAATCTAGCA	58	110
	Antisense: AATGCAGAGGTGGTTTCACTCA		
Sox9	Sense: AGCCGAAAGCGGAGCTCGAAACT	58	215
	Antisense: GCACTTAGGAAGGCGCGGGGT		
Col2A1	Sense: CAGGATGGGCAGAGGTAT	58	109
	Antisense: CGTCTTCACAGATTATGTCGT		
GAPDH	Sense: GACTTCAACAGCAACTCCCAC	60	125
	Antisense: TCCACCACCCTGTTGCTGTA		

### Statistical analyses

Data are mean ± standard deviation (SD). Statistical analyses were performed with the GraphPad Prism 5 software (GraphPad Software Inc., CA, USA). Unpaired t-test was applied to assess the difference between adipocytes size of macrodactyly and control tissues. Student’s t-test was applied to assess cell proliferation and cell cycle data. The differences among multiple groups in gene expression patterns of adipogenic, osteogenic and chondrogenic induction were evaluated by two-way ANOVA. P < 0.05 was considered statistically significant.

### Data Availability

No datasets were generated or analysed during the current study.

### Ethics, consent and permissions

This study was approved by the ethics committee of Shanghai 9th People’s Hospital (reference: 201580). Informed consent was obtained from all patients, or legal parents or guardians of children. All methods were performed in accordance with the relevant guidelines and regulations.

### Consent to publish

We have obtained consent from all patients, or legal parents or guardians of children, to report and publish individual patient data.
